# An Update on the Prognostic and Predictive Serum Biomarkers in Metastatic Prostate Cancer

**DOI:** 10.3390/diagnostics10080549

**Published:** 2020-07-31

**Authors:** Helen Saxby, Christos Mikropoulos, Stergios Boussios

**Affiliations:** 1 St Luke’s Cancer Center, Royal Surrey County Hospital, Egerton Rd, Guildford GU2 7XX, UK; h.saxby@nhs.net (H.S.); christos.mikropoulos@nhs.net (C.M.); 2 Medway NHS Foundation Trust, Windmill Road, Gillingham, Kent ME7 5NY, UK; 3 AELIA Organization, 9th Km Thessaloniki-Thermi, 57001 Thessaloniki, Greece

**Keywords:** serum biomarkers, metastatic prostate cancer, microRNA biomarkers, bone metabolism biomarkers, neuroendocrine biomarkers, metabolite biomarkers

## Abstract

Serum biomarkers are molecules produced by normal and abnormal cells. Prostate specific antigen (PSA) is an example of a serum biomarker used widely in the diagnosis and prognostication of prostate cancer. PSA has its limitations as it is organ- but not cancer-specific. The aim of this review is to summarize the current published data on the potential prognostic and predictive biomarkers in metastatic prostate cancer (mPC) that can be used in conjunction with PSA. These biomarkers include microRNAs, androgen receptor variants, bone metabolism, neuroendocrine and metabolite biomarkers, and could guide treatment selection and sequence in an era where we strive to personalized therapy.

## 1. Introduction

Prostate cancer is the second most commonly diagnosed cancer in males worldwide and the fifth most frequent cause of cancer related death in men worldwide. It is a heterogenous condition ranging from relatively indolent to aggressive disease. Androgen deprivation therapy is the mainstay of treatment in metastatic prostate cancer (mPC), specifically using luteinising hormone releasing hormone agonists or antiandrogens. Unfortunately, prostate cancer cells become resistant to this treatment (termed castration-resistant), usually after 18–24 months [1,2,3,4].

Circulating serum biomarkers are molecules produced by normal and abnormal cells. They are attractive for clinical use as they are readily available and easily obtained through minimally invasive methods [5]. Prostate specific antigen (PSA) is an example of a serum biomarker used widely in the diagnosis and prognostication of prostate cancer and in assessing response to treatment. It is a kallikrein protease produced by the luminal cells in the prostate gland. PSA has its limitations as it is organ- but not cancer-specific. It can be elevated in benign conditions, including prostatitis and benign prostatic hyperplasia (BPH). At present, PSA in addition to the Gleason score and radiological staging are used in the diagnosis and prognostication of prostate cancer [6,7,8]. Novel biomarkers are needed in prostate cancer to improve the prognostic and predictive accuracy of PSA, imaging and biopsy alone [9,10].

There is an expanding choice of treatments for mPC; predictive factors would help clinicians to tailor the type and sequence of treatment to each patient. Despite extensive research, very few biomarkers have been implemented in routine clinical practice to date [11,12].

The aim of this review is to summarise the current published data focussing on a selection of promising biomarkers with the potential to improve the prognostic and predictive accuracy of PSA in mPC. The categories of biomarkers investigated include microRNA, androgen receptor variants (AR-Vs), bone metabolism, neuroendocrine and metabolite biomarkers [3].

## 2. Methods

**Figure 1:**
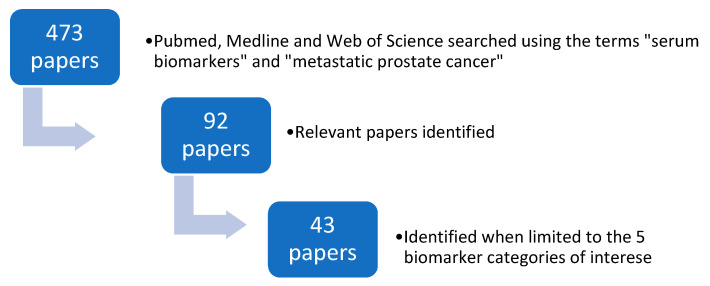
The search strategy used to identify relevant papers.

Pubmed, Medline and Web of Science were searched using the search terms “serum biomarkers” and “metastatic prostate cancer”. The search period was from April 2010 to April 2020. Four hundred and seventy papers were identified when filtered using the terms “humans” and “English” language. Ninety-two relevant papers were identified from this list. Forty-three papers were then identified when limited to the five biomarker categories of interest (Figure 1).

## 3. Results and Discussion

PSA does not provide a prediction of disease outcome in newly diagnosed prostate cancer patients and cannot determine the course of treatment. On the other hand, there have been rapid achievements in the treatment of prostate cancer. The role for biomarkers to select patients that may benefit from a particular therapy is crucial. MicroRNAs (miRNAs), androgen receptor variants (AR-Vs), bone metabolism, neuroendocrine and metabolite biomarkers are promising candidates that will allow the precision medicine revolution to take place.

### 3.1. MicroRNA Biomarkers

At present, there is significant interest in the use of miRNAs as biomarkers to improve the diagnosis and prognostication of prostate cancer. miRNAs are endogenous short non-coding RNAs (19–26 nucleotides in length) that bind to complementary messenger RNA to suppress gene expression post transcriptionally. Approximately 1000 human miRNAs have been identified to date. Alterations in miRNA expression can affect important cellular processes, including the cell cycle, proliferation, apoptosis and epithelial to mesenchymal transition (EMT) [13,14,15]. They are relatively stable biomarkers and can be found free in the serum and within exosomes (extracellular vesicles in the serum, semen and urine). miRNAs are found in greater concentration in exosomes, as they are protected against RNAases. Reliable methods to extract miRNAs from serum and exosomes have been developed and documented. Reverse transcriptase polymerase chain reaction is then used to quantify miRNAs [16,17,18].

miRNAs can be divided into oncogenes or tumour suppressors dependent on their targets. Oncogenic miRNAs in prostate cancer can enhance proliferation by down regulating cell-cycle-dependent kinase inhibitors and transcription factors [19]. Brase et al. screened 667 miRNAs in serum samples from patients with metastatic and localised prostate cancer. Sixty-nine miRNAs were detected in significantly higher concentrations in the serum of patients with mPC, when compared to patients with localized prostate cancer. miRNA-375 was the strongest marker and was also significantly associated with lymph node involvement and metastases, but not the Gleason grading of prostate cancer. miRNA-375 regulates genes involved with cell growth and proliferation in the pancreas; its function is essential for glucose-induced insulin secretion. Further research is required to establish its function in prostate cancer [13]. A further study by Nguyen et al. also found that serum miRNA-375 in addition to miRNA-378 and miRNA-141 were significantly upregulated in castrate-resistant prostate cancer (CRPC), compared to localized prostate cancer. miRNA-141 plays an essential function in epithelial-to-mesenchymal transition but little is known with regards to the function of miRNA-378 [20].

High levels of miRNA-21 have been observed in multiple different cancers. In prostate cancer, it contributes to pathogenesis and castration resistance. Zhang et al. demonstrated higher serum concentrations of miRNA-21 in CRPC than hormone sensitive prostate cancer (HSPC) [17]. Levels of miRNA-21 were higher in CRPC who were resistant to docetaxel chemotherapy than in those that were sensitive. It was hypothesized that miRNA-21 plays a role in the transformation of hormone sensitive to castrate-resistant disease. Measuring the plasma concentration of miRNA-21 in addition to the PSA has been shown to improve the specificity and sensitivity of detecting localized prostate cancer from healthy controls and patients with BPH. Adding miRNA-21 concentration to PSA improved the sensitivity and specificity of detecting patients with mPC [21].

Further studies have been performed to determine whether miRNAs can determine which patients with CRPC will have a favourable response to treatment with docetaxel chemotherapy. This would be of great clinical benefit, as there is only a 50% response rate in all patients with CRPC treated with docetaxel and the treatment can cause significant side effects. Serum miRNAs in the miRNA-200 and miRNA-17 families were associated with a PSA response and improved overall survival (OS) in CRPC receiving treatment with docetaxel. They have the potential to be used as early treatment response and prognostic biomarkers. The miRNA-200 family members are involved in the regulation of EMT, which is a mechanism of drug resistance and metastasis, whereas the miRNA-17 family has immune regulatory functions. These mi-RNA families may be involved in the mechanism of docetaxel resistance [22].

Exosomal-miRNA-1246 is a further potential miRNA biomarker with predictive and prognostic potential. Higher exosomal-miRNA-1246 expression was found in prostate cancers with higher stage, grade, presence of positive lymph node, and distant metastases. However, the serum concentration of miRNA-1246 is lower than healthy normal. Bhagirath et al. suggested that miRNA-1246 is a prostate cancer tumour suppressor miRNA and inhibits EMT, cellular proliferation, and survival, and promotes apoptosis [16]. In a subsequent report, it was found that miRNA-4288 was also downregulated in prostate cancer [23]. Interestingly, it was found to be related to the increasing tumour grade and high serum PSA in Caucasians but not in African Americans, who are known to have a higher prostate cancer incidence and mortality rate. This supports the idea that prostate cancer derives from different molecular pathways in different races.

An elevated level of miRNA-141 correlates with an increasing number of bone metastases [24]. Further evidence suggests that miRNA-218-5p could also be a biomarker for bone metastases. It suppresses the NF-κB signaling pathway, which suppresses the invasion and migration ability of prostate cancer cells. MiRNA-218-5p expression was reduced in the serum of patients with bone metastatic prostate cancer compared to those with non-bone metastatic prostate cancer. Serum levels were significantly correlated with PSA levels, Gleason grade and bone metastasis status, and low levels of miRNA-218-5p predicted poor bone metastasis-free survival [25].

### 3.2. Androgen Receptor Variants (AR-Vs)

CRPC is not androgen independent; however, 20–40% of patients have a primary resistance to novel antiandrogens (enzalutamide and abiraterone) and nearly all develop a secondary resistance. AR-Vs are a possible cause of this resistance. They are truncated androgen receptor (AR) proteins without the AR-ligand binding domain. This results in constitutive AR signaling in the absence of androgens. The AR Splice Variant 7 (AR-V7) has been studied in detail to date. It can be detected in circulating tumour cells, exosomes and whole blood samples. Patients with metastatic castrate-resistant prostate cancer (mCRPC), who are receiving enzalutamide or abiraterone and are positive for AR-V7, have a worse progression free survival (PFS) and OS than patients who are negative for AR-V7. However, being positive for AR-V7 is not associated with a significant resistance to taxanes. AR-V7 could be a useful biomarker in predicting patients, who will benefit from treatment with novel androgen receptor blocking agents [26,27,28].

### 3.3. Bone Metabolism Biomarkers

Prostate cancer frequently metastasizes to the bone. Over 90% of patients with mPC have bone metastases, which can cause considerable morbidity, including severe bone pain, hypercalcaemia, spinal cord compression and pathological fracture [29]. Being able to predict patients who are at higher risk of developing these skeletal related events (SRE) enables optimisation of their clinical management to improve their quality of life and possibly survival. Currently, bone metastases are evaluated by nuclear medicine bone scans. These scans have a low specificity for detecting early bone metastases [5,30,31].

Bone metastases from prostate cancer are typically sclerotic in nature. Tumour cells secrete factors, which stimulate osteoblasts and therefore bone formation. Biochemical markers of bone metabolism include bone alkaline phosphatase (BALP), N-terminal propeptide of type 1 collagen (P1NP), beta-isomer of carboxyterminal telopeptide of collagen 1 (β-CTX), bone sialoprotein (BSP) and osteopontin (OPN). BALP and P1NP are markers of bone formation, whereas β-CTX is a marker of bone resorption. OPN and BSP are markers of both bone formation and resorption. These markers are all present in the serum and enable the monitoring of osteoclast and osteoblast activity [32].

BALP is an enzyme secreted by osteoblasts that promotes bone mineralization. Increased levels in the serum are a sign of increased osteoblast activity. It can also be elevated due to hepatic disease, as monoclonal antibodies against BALP exhibit 15% cross-reactivity with a hepatic isoenzyme [31,32]. Type 1 collagen is a significant component of the bone matrix, and forms from type 1 procollagen. P1NP is a propeptide cleaved from the N-terminal of procollagen and can be detected in the serum. Levels of P1NP are associated with bone formation [31]. Type 1 collagen releases carboxy-telopeptides (CTX) during bone resorption into the serum and urine. β-CTX is an isomerised form of CTX and has a circadian rhythm depending on eating. It should therefore be tested early in the morning in patients who have fasted [30,31]. BSP and OPN are protein components of the non-collagenous bone matrix. BSP is expressed in osteoclasts and osteoblasts. OPN is expressed in osteoblasts. They are involved in the regulation of bone resorption and formation as well as in the process of forming metastases [31].

Several studies have demonstrated that markers of bone metabolism are useful in predicting SRE and prognostication in patients with prostate cancer and bone metastases [5,32,33,34]. Sonpavde et al. performed a retrospective analysis of 601 patients with CRPC selected for the TAX327 trial to assess the ability of total alkalkine phosphatase (tALP) to predict survival [35]. In patients with a baseline tALP ≥120 IU/L, normalisation of tALP by day 90 following treatment with docetaxel or mitoxanthrone was associated with a better median OS, whereas an increase in tALP by day 90 was associated with significantly poorer OS.

Jung et al. demonstrated an association between CTX and P1NP, and SRE and OS, in patients with prostate cancer and bone metastases treated with zolendronic acid (ZA) [34]. In TUGAMO prospective multicentre study, the serum levels of BALP, P1NP and β-CTX were monitored at baseline and then three-monthly in 98 patients with prostate cancer and bone metastases over an 18-month time period. All patients received ZA 4 mg every four weeks. Once ZA was established, no patients received hormone therapy, which can cause a rise in the bone metabolism biomarkers. Alternative standard systemic anti-cancer therapies were allowed during this study. All patients had raised markers at baseline, and a significant positive correlation was demonstrated between the level of the markers and the burden of bone metastases. No association was detected between the bone biomarkers and disease progression. A decrease in BALP and P1NP were strongly associated with the development of SRE. A decrease in BALP of <87% between baseline and 3 months indicated a 4-fold increase risk of SRE. A lack of normalisation of P1NP after treatment increased the risk of SRE 3.8 times. A decrease in β-CTX was associated with a lower OS (a decrease of <40% between baseline and 3 months indicated a six-fold increase in mortality) [32].

BSP and OPN have demonstrated use in the prognostication in prostate cancer. Higher BSP levels are related to a shorter time to develop bone metastases in patients with prostate cancer [36]. OPN is not a marker of tumour burden but may be of use in assessing treatment response post chemotherapy in patients with CRPC [6].

### 3.4. Neuroendocrine Biomarkers

Neuroendocrine differentiation (NED) represents a poor prognostic feature and is generally seen in higher-grade and higher-stage prostate cancers. It is reported that 10 to 100% of conventional prostate adenocarcinomas display evidence of NED immunohistochemically, with a higher incidence detected in castrate resistant disease. Neuroendocrine prostate cancer (NEPC) is uncommon and is associated with low PSA secretion and loss of androgen receptor expression. De novo small cell prostate cancer is an aggressive histological variant and is very rare in untreated patients (<1%) [37,38].

Neuroendocrine cells store neuropeptides in cytoplasmic granules, including chromogranin A (CgA) and neurone-specific enolase (NSE); the most widely evaluated neuropeptides in prostate cancer. Neuropeptides may stimulate growth, differentiation and secretory processes, and can be detected in the serum [38]. Serum levels of CgA and NSE are associated with the degree of NED in prostate cancer cells. Serum levels of CgA are significantly higher in metastatic than in non-metastatic prostate cancer, and are adversely associated with survival in patients with CRPC and a Gleason score ≥8 [39,40,41].

Conteduca et al. evaluated serum CgA levels in predicting patient outcome in a retrospective study [42]. Thirty five patients with metastatic castrate-resistant prostate cancer who were starting treatment with enzalutamide having progressed on docetaxel chemotherapy had baseline serum CgA measurements. These patients were subdivided into three groups; group A: normal serum CgA levels <120 ng/mL, group B: serum CgA level within three times the upper limit of normal (≥120 ng/mL to <360 ng/ml) and group C: serum CgA level over three times the upper limit of normal ≥ 360ng/ml. A serum CgA level over three times the upper limit of normal (group C) was significantly associated with a poorer median PFS (2.5 months vs. 4.7 months in group A, *p* = 0.0301) and median overall survival (group A did not reach median OS, median OS was 9.4 months in group B, and 3.4 months in group C, *p* = 0.0011). Further retrospective studies have also demonstrated a significant association between baseline serum CgA over three times the upper limit of normal and early radiological disease progression in patients with mCRPC treated with abiraterone after receiving docetaxel chemotherapy [43,44,45].

No relationship has been observed between baseline CgA and PSA response. CgA may be expressed by a subclone of prostate cancer cells in which NED affects clinical outcome without affecting the PSA response, i.e., NED represents an alternative AR independent mechanism of resistance [37,42,43,46].

Fan et al. evaluated the benefit of baseline serum CgA and NSE in guiding the sequence of treatments in patients with mCRPC [47]. The serum levels of CgA and NSE in 88 patients with mCRPC were collected retrospectively before commencing first-line treatment with either docetaxel or abiraterone. Patients with an elevation of at least one of the neuroendocrine markers had a modest but significantly better OS if they received sequential treatment with docetaxel and then abiraterone rather than abiaterone followed by docetaxel (21.7 months vs. 19.9 months, *p* = 0.035). There was no difference in OS and sequence of treatment in patients who did not have an elevated neuroendocrine marker at baseline.

### 3.5. Metabolite Biomarkers

Metabolic changes are affected by environmental, genetic and epigenetic factors. Metabolomic profiling of prostate cancer has been a promising area in the development of novel biomarkers. The prostate produces extremely high amounts of citrate, and citrate metabolism differs in prostate cancer compared to BPH [48].

The European Prospective Investigation into Cancer and Nutrition (EPIC) was a large prospective trial published in 2017 investigating 122 serum metabolites and their association with prostate cancer risk [49]. Seven metabolites were associated with an increased risk of prostate cancer; however, these were not significant when a correction for multiple testing was applied. The strongest association was detected with a glycerophospholipid phosphatidylcholine.

Subsequently, Huang et al. conducted a large prospective serum metabolomic analysis of lethal prostate cancer in 523 cases and 523 controls [50]. Thirty-four serum metabolites were associated with death from prostate cancer. An inverse association was detected between serum oxidative stress-related thioproline, cysteine and cystine, and lethal prostate cancer, whereas higher levels of leucylglycine and several gamma-glutamyl amino acids were associated with a higher risk of lethal prostate cancer. Cases with mPC at diagnosis were associated with elevated serum fatty acid metabolites and ketone bodies. This could also be related to a western diet.

The metabolite sarcosine (an N-methyl derivative of the amino acid glycine) is found in significantly higher concentrations in mPC than in non-mPC. It is thought to promote prostate cancer growth and progression, as in vitro studies demonstrated that administering exogenous sarcosine to benign prostate cells induced an invasive phenotype. Sarcosine has proven to be an independent prognostic factor in terms of PFS and OS in prostate cancer [51,52].

Serum glutamate levels were positively correlated to Gleason grade and prostate cancer aggressiveness in a study by Koochekpour et al. [48]. Higher serum levels of glutamate in patients with prostate cancer can be explained by an increased rate of glutaminolysis in proliferating prostate cancer cells or carboxypeptidase function of prostate specific membrane antigen (PSMA) overexpressed by prostate cancer cells.

Higher concentrations of serum methionine metabolites (homocysteine, cystathionine and cystine) at the time of radical prostatectomy were predicitive of early biochemical recurrence and aggressiveness of disease in a study conducted by Stabler et al. [53].

Table 1 depicts the prognostic and predictive serum biomarkers in metastatic prostate cancer that have been reported in the literature.

## 4. Conclusions and Future Perspectives

The current risk stratification tools used in prostate cancer are inadequate. Additional serum biomarkers can potentially identify patients with more aggressive disease and enable the most effective treatments to be tailored to specific patients in the optimum sequence. This review highlights five different classes of promising biomarkers: miRNA, AR-Vs, bone metabolism, neuroendocrine and metabolic markers. They are all detected in the serum and are practical and minimally invasive to obtain. These biomarkers have been extensively investigated over the last decade, however none are routinely used in clinical practice. Further prospective trials are required for clinical validation.

In addition to the aforementioned serum biomarkers, genomic profiling of prostate cancer is an exciting emerging field. Evidence is accumulating associating specific genomic alterations with castrate resistance and metastatic progression. Genomic biomarkers that have been investigated in clinical trials include *BRCA1*/*BRCA2*/*ATM* for PARP inhibitors, *PTEN*/*AKT* for AKT inhibitors and *PIK3CB* for (PI3K)-β inhibitors. Genomic profiling could add further important predictive and prognostic information when managing patients with prostate cancer. Machine learning is a field that has developed with advances in computing and artificial intelligence. Machine learning models can rapidly analyse large data sets, and their use in identifying biomarkers from genomic data in prostate cancer is promising and attracting much research [54,55].

## Figures and Tables

**Table 1:** Potential prognostic and predictive serum biomarkers in metastatic prostate cancer.

Prognostic and Predictive Serum Biomarkers in Metastatic Prostate Cancer	
microRNAs	miRNA-17 miRNA-21 miRNA-141 miRNA-200 miRNA-218-5p miRNA-375 miRNA-378 miRNA-1246 miRNA-4288
Androgen receptor variants	AR-V7
Bone metabolism markers	Alkaline phosphatase (ALP) Procollagen-1 N-terminal peptide (P1NP) Beta-C-terminal telopeptide (β-CTX) Bone sialoprotein (BSP) Osteopontin (OPN)
Neuroendocrine markers	Chromogranin A (CgA) Neurone-specific enolase (NSE)
Metabolites	Sarcosine Methionine metabolites Glutamate
